# Morphological Characterisation of Three Populations of *Heterobranchus longifilis* from Nigeria

**DOI:** 10.21315/tlsr2024.35.1.9

**Published:** 2024-03-30

**Authors:** A. Alih Raphael, Solomon Shola Gabriel, Olufeagba Samuel Olabode, O. Cheikyula Joseph, Abol-Munafi Ambok Bolong, Mhd Ikhwanuddin, Okomoda Victor Tosin

**Affiliations:** 1 Department of Fisheries and Aquaculture, College of Forestry and Fisheries, Joseph Sarwan Tarka University Makurdi (Formerly known as University of Agriculture, Makurdi), P.M.B. 2373, Makurdi, Nigeria; 2 Institute of Tropical Aquaculture and Fisheries (AKUATROP), Universiti Malaysia Terengganu, 21030 Kuala Nerus, Terengganu, Malaysia; 3 Faculty of Food Science and Fisheries, Universiti Malaysia Terengganu, 21030 Kuala Nerus, Terengganu, Malaysia; 4 STU-UMT Joint Shellfish Research Laboratory, Shantou University, Shantou, 515063 Guangdong Province, China

**Keywords:** Meristic Count, Morphological Plasticity, Catfish, Population, Kiraan Meristik, Keplastikan Morfologi, Ikan Keli, Populasi

## Abstract

This study attempted to discriminate the population of *Heterobranchus longifilis* in Nigeria using their morphological characteristics. Therefore, 60 sexually mature wild samples of *H. longifilis* (1:1 for the male and female ratio) of relatively similar size (40 cm) were collected from three eco-regions namely, Guinea Savanna (Benue River, Makurdi), Rainforest Savanna (Niger River, Onitsha) and Sahel Savanna (Rima River, Sokoto). They were transported to the hatchery unit of the Fisheries and Aquaculture Department, Joseph Sarwan Tarka University Makurdi where the morphometric data was collected. The data for 39 traditional morphometric measurements and 5 meristic counts obtained from each fish were subjected to univariate and multivariate analysis. While significant differences were observed in some parameters following univariate analysis; it was revealed that the morphometric parameters and meristic counts could not separate the fish from the different ecoregions into distinct multivariate spaces or clusters following Principal Component Analysis. Hence, this suggests that morphological parameters cannot be used to discriminate *H. longifilis* from the different ecoregions. Studies using molecular markers are needed to further characterise the distinctiveness of the different populations.

## Abstract (ms)

Kajian ini cuba membezakan populasi *Heterobranchus longifilis* di Nigeria menggunakan ciri morfologi mereka. Oleh itu, 60 sampel *H. longifilis* liar yang matang secara seksual (1:1 untuk nisbah jantan dan betina) dengan saiz yang agak serupa (40 cm) telah dikumpulkan dari tiga kawasan eko iaitu, Guinea Savanna (Sungai Benue, Makurdi), hutan hujan Savanna (Sungai Niger, Onitsha) dan Sahel Savanna (Sungai Rima, Sokoto). Sampel telah dibawa ke unit penetasan Jabatan Perikanan dan Akuakultur, Universiti Joseph Sarwan Tarka Makurdi di mana data morfometrik dikumpulkan. Data untuk 39 ukuran morfometrik tradisional dan 5 kiraan meristik yang diperolehi daripada setiap ikan telah dibahagikan kepada analisis univariat dan multivariat. Walaupun perbezaan ketara diperhatikan dalam beberapa parameter berikutan analisis univariat; telah didedahkan bahawa parameter morfometrik dan kiraan meristik tidak dapat memisahkan ikan daripada Eko kawasan yang berbeza ke dalam ruang atau gugusan multivariat yang berbeza berikutan Analisis Komponen Utama. Oleh itu, ini menunjukkan bahawa parameter morfologi tidak boleh digunakan untuk membezakan H. longifilis daripada Eko kawasan yang berbeza. Kajian menggunakan penanda molekul diperlukan untuk mencirikan lagi keunikan populasi yang berbeza.

**Highlights**

- Univariate analysis of morphometric data shows significant differences among strains.
- Principal component analysis (PCA), revealed complete overlap of the fish of the different strain.
- The morphometric parameters and meristic counts cannot be used to separate the different strains into distinct multivariate clusters.

## INTRODUCTION

The genus *Heterobranchus* is one of the most important freshwater fish genera in sub-Saharan Africa (Ataguba *et al*. 2009). However, on-like the genus *Clarias* which has been extensively studied at various levels under different aquaculture disciplines around the world, research on the species of the genus *Heterobranchus* is still localised within the environs of Africa (Alih *et al*. 2022). Although, several fundamental studies earlier conducted on species such as *H. longifilis* have led to the improvement of breeding, and aquaculture production under captivity (Baras 1999; Poncin *et al*. 2002; Ataguba *et al*. 2009; Olufeagba *et al*. 2016; Okomoda *et al*. 2017); there are still research gaps as regards to information about morphological variation between the different population of the fish. Such studies in conjunction with genetic characterisation can be the basis upon which a selective breeding program could be conducted to improve the cultural performance of its progenies (Solomon *et al*. 2015).

According to Normala *et al*. (2017), conventional morphological methods continue to have an important role in stock identification despite the development of advanced techniques that can directly examine biochemical or genetic variations. Morphological and biometrical characteristics remain the simplest and most rapid methods used in delineating, discriminating and classifying fish stocks/identifying species (Hockaday *et al*. 2000; Turan *et al*. 2004). The study of the variability of morphological characters of fish is important to inform subsequent genetic studies (Olufeagba *et al*. 2015a, 2015b; Okomoda *et al*. 2018). The phenotypic plasticity of a population can therefore be used to distinguish between different stocks (O’Reilly & Horn 2004). This is because it is largely not under genetic control alone but influenced by environmental conditions (Chittenden *et al*. 2010; Arora & Julka 2013).

Fish population responses to environmental changes involve the modification of physiological and behavioural characteristics. This consequently affects the morphological and reproductive status as the fish attempts to mitigate the effect of the environmental changes experienced (Turan 1999). This means the characteristics of the different populations are shaped/impacted by the environmental conditions where each population lives. Thus, genetic information alone is not sufficient in choosing the base population for a selective breeding program but an interaction between genetics and environmental factors which are expressed in different morphological changes (Scheiner & Callahan 1993; Hoffman & Merila 1999). Since different habitat experiences different environmental changes, therefore different impacts are experienced by different population structures (Ahmad 2015). These habitat-specific environmental conditions may include such factors as predation pressure, food availability, salinity, temperature, turbidity and water condition (Scapini *et al*. 1999; Maltagliati *et al*. 2003; Remerie *et al*. 2005). The current study was therefore designed to determine the extent of morphological variation of three populations of *H. longifilis* from three eco-regions in Nigeria. This is considered the first step toward developing a selective breeding program for fish species in captivity (Okomoda *et al*. 2022).

## MATERIALS AND METHODS

**Figure 1:**
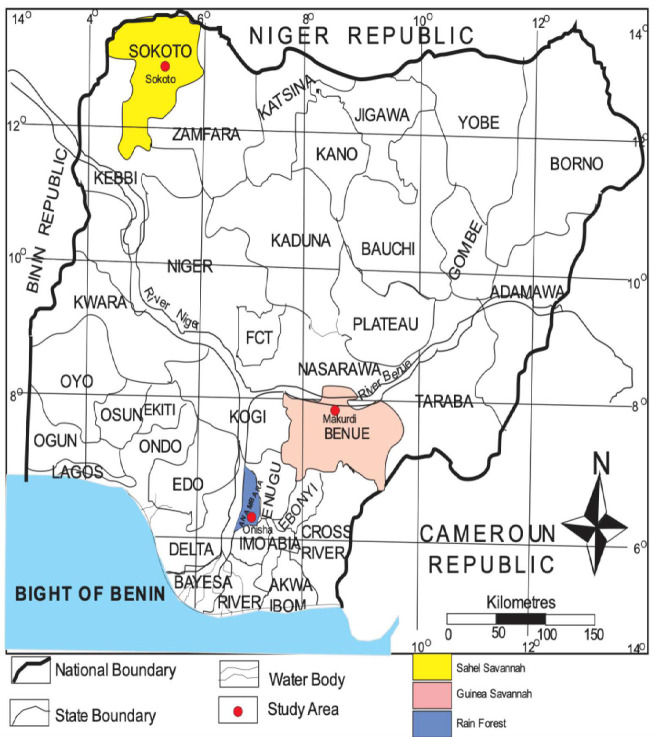
Map of the study area (Source: Ministry of Land and Survey Makurdi)

The populations of *H. longifilis* for this study were obtained from three eco-regions of Nigeria (Fig. 1). The first is the Sahel Savanna precisely from Rima River, in Sokoto State located at latitude 13.0059°N, longitude 5.2476°E with an annual mean temperature of 28.30°C and rain ranges between 500 mm and 1,300 mm. *H. longifilis* samples were also gotten from the Guinea Savanna at the Benue River in Makurdi, Benue State located at latitude 7.7322°N, longitude 8.5391°E. This region has a mean annual temperature of 26.7°C and a mean annual rainfall of 1,077 mm. The place for the fish collection was the Rainforest region at the Niger River, in Onitsha with latitude 6.1329°N and longitude 6.7924°E. It has an annual mean temperature of 27.0°C and a mean annual rainfall of 1,828 mm (https://en.climate-data.org).

A total of 60 fish samples of reproductive age (i.e., 1:1) and relatively similar sizes of about 40 cm were collected from each eco-region for two months (i.e., the collection was done weekly). Hence, a total of 180 fish samples were collected from the three eco-regions. The fish samples were identified using the identification keys adopted by Moses and Olufeagba (2009) following confirmation from the local fishermen before transporting live from the eco-regions to Makurdi in 50 L open black plastic jerry-cans equipped with continuous aeration (using battery-powered motors). Upon reaching the research farm, morphological parameters were determined as shown in the next section.

### Morphometric and Meristic Measurements

The morphometric and meristic characterisation in the current study were according to the previous method described by Okomoda *et al*. (2018). Data for 39 morphometric measurements and 5 meristic counts were taken from each sample of *H. longifilis* collected from the three eco-regions of Nigeria, using a measuring board, meter rule, and a weighing balance. This includes body weight (BW measured in grams), head length (HL), standard length (SL), total length (TL), dorsal fin length (DFL), adipose fin length (ADFL), head width (HW), eye diameter (ED), inter-orbital distance (IOD), body depth (BD), occipital fontanelle length (OFL), occipital fontanelle width (OFW), vomerine width (VW), dorsal fin height (DFH), predorsal length (PDL), pectoral fin length (PeFL), prepectoral length (PPcL), pelvic fin height (PFH), pelvic fin length (PFL), anal fin ray number (AFRN), anal fin length (AFL), pelvic fin to anal fin (PvFAF), caudal fin ray number (CFRN), snout length (SnL), nasal barbell length (NBL), maxillary barbell length (MxBL), premaxillary length (PmxL), vomerine length (VL), vomerine gap (VG), dorsal fin ray number (DFRN), anal fin height (AFH), caudal fin length (CFL), caudal fin height (CFH), and caudal peduncle depth (CPdD).

### Data Analysis for Morphological Parameters

The measurements taken were first standardised to remove the effect of size before analysis was done following the method adopted by Murta *et al*. (2008) and Jaferian *et al*. (2010). By doing so, the individuals from each sample collection were normalised into a single arbitrary size, while maintaining individual variation within the sample (Sen *et al*. 2011). The relation used to achieve this was the allometric formula described by Elliott *et al*. (1995):


$$M_{adj}=M{\left(\frac{L_{s}}{L_{o}}\right)}^{b}$$

where *M* = observed character measurement, *M_adj_* = size-adjusted measurement, *L_o_* = standard length of the fish, *L_s_* = overall mean of the TL for all the progenies, and *b* = estimated for each character from the collected data as the slope of the regression of log *M* on log *L_o_*, using all fish of all the progenies.

Upon transforming the data, only data from 162 individuals of the 180 samples collected were used for the multivariate analysis of the Principal Component Analysis (PCA) using PAST free software. The exclusion of some samples from the analysis was due to incompleteness in the data entry due to human errors. The PAST free software was also used to obtain a sample centroids graph on the biplot, which then allows the determination of the most valuable morphological character that can be used to separate the fish groups into distinct multivariate spaces. Dendrograms with complete linkage and Euclidean distances of the fishes were also determined using the PAST free software and reported accordingly.

## RESULTS

The result of the present study based on the univariate analysis of variance (ANOVA) reveals similarities in most of the morphometric parameters except for 13 parameters namely: CPL, PrPel, PFW, PFDT, PSL, PFL, DFPF, LML, VW, SnL, PrML, MBL and NBL (Table 1). The Onitsha population had higher values in 9 of the 13 parameters (*P* < 0.05) while in most cases, Makurdi and Sokoto were similar and had the least values. Expressing the morphometric parameters as percentages of standard length, however, did not change the trend of the result shown in Table 2. The result for the PCA for the transformed homologous morphometric parameters of the *H. longifilis* (Table 3) only used the first three principal components (PC), as the eigenvalue was more than 1. This is according to the recommendations earlier made by Kaiser (1961).

**Figure 2:**
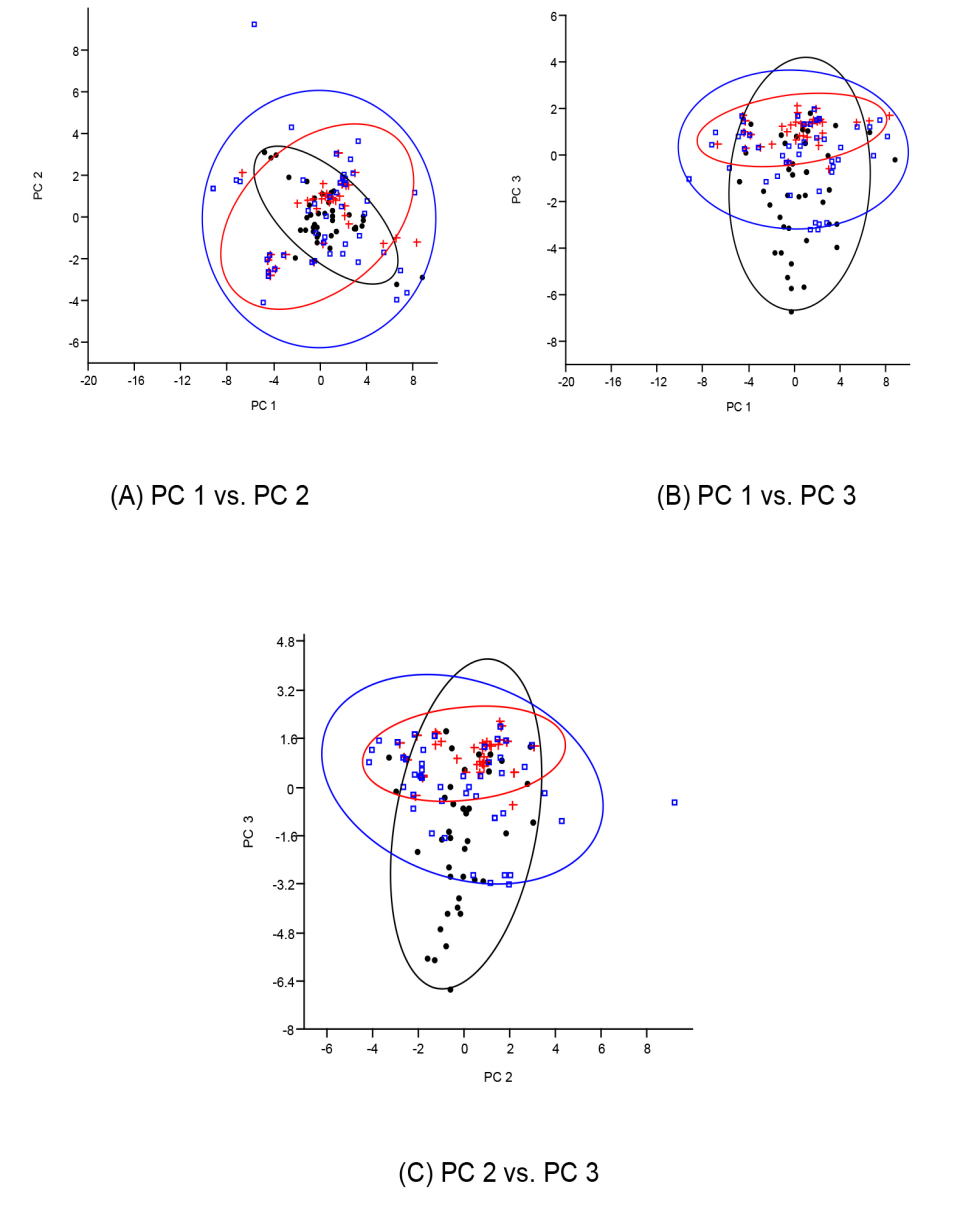
Principal component analysis of transformed morphometric measurements of *H. longifilis* from three geographical locations in Nigeria. The biplot shows individual fish scores. Dot = Makurdi; Red = Onitsha; Blue = Sokoto.

**Figure 3:**
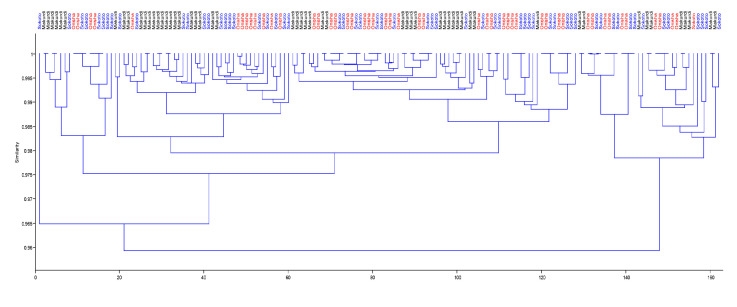
Dendrogram with complete linkage and euclidean distance for morphometric parameter of *H. longifilis* from three geographical locations in Nigeria. The biplot shows individual fish scores. Black label = Makurdi; Red label = Onisha; Blue label = Sokoto.

For the morphometric parameters, the first principal component (PC1) accounted for 28.49% of the total variance with only negative coefficients. The second principal component (PC2) had a mix of positive and negative coefficients and counted for 9.43% of the total variance in this study. In the same vein of mixed positive and negative coefficients, PC3 accounted for just 8.33% of the total variation. In summary, all three principal components accounted for only 46.25% of the variance observed for the *H. longifilis* from the ecoregions. The low cumulative variance did not permit the recommendation of influential variables as the different populations could not be separated into unique multivariate spaces as seen in the biplot in Fig. 2. Similarly, the dendrogram of complete linkage and Euclidean distance shown in Fig. 3 also showed multiple overlaps of the samples from the different ecoregions.

**Figure 4:**
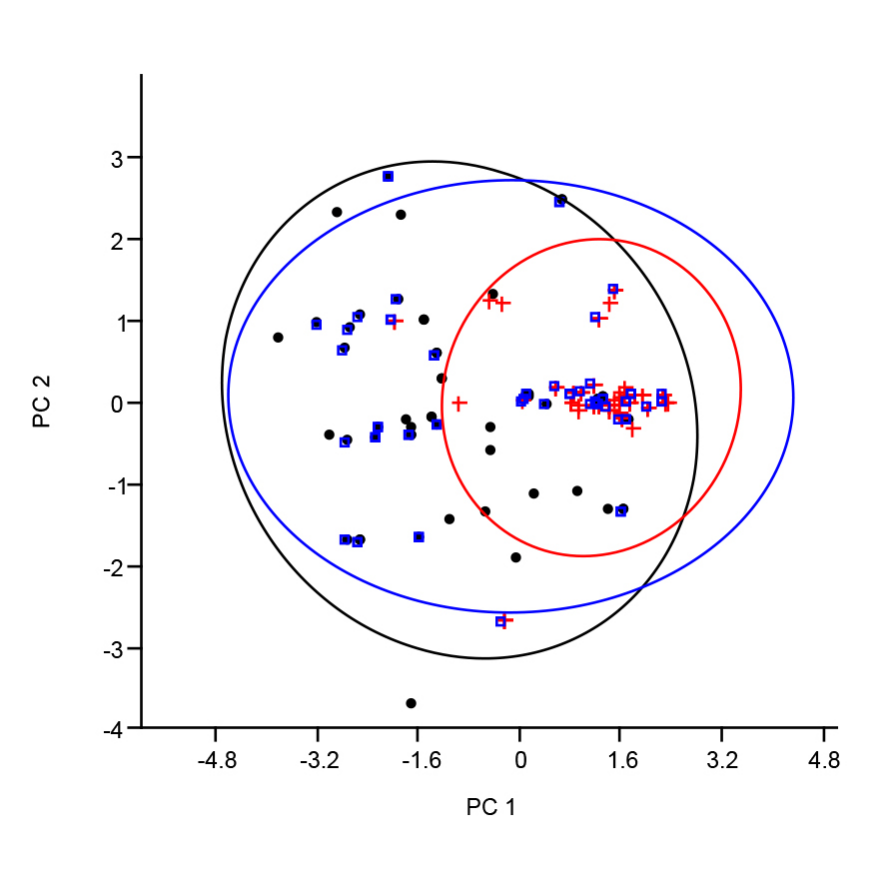
Principal component analysis of meristic count of *H. longifilis* from three geographical locations in Nigeria. The biplot shows individual fish scores. Dot = Makurdi; Cross = Onitsha; Square = Sokoto.

**Figure 5:**
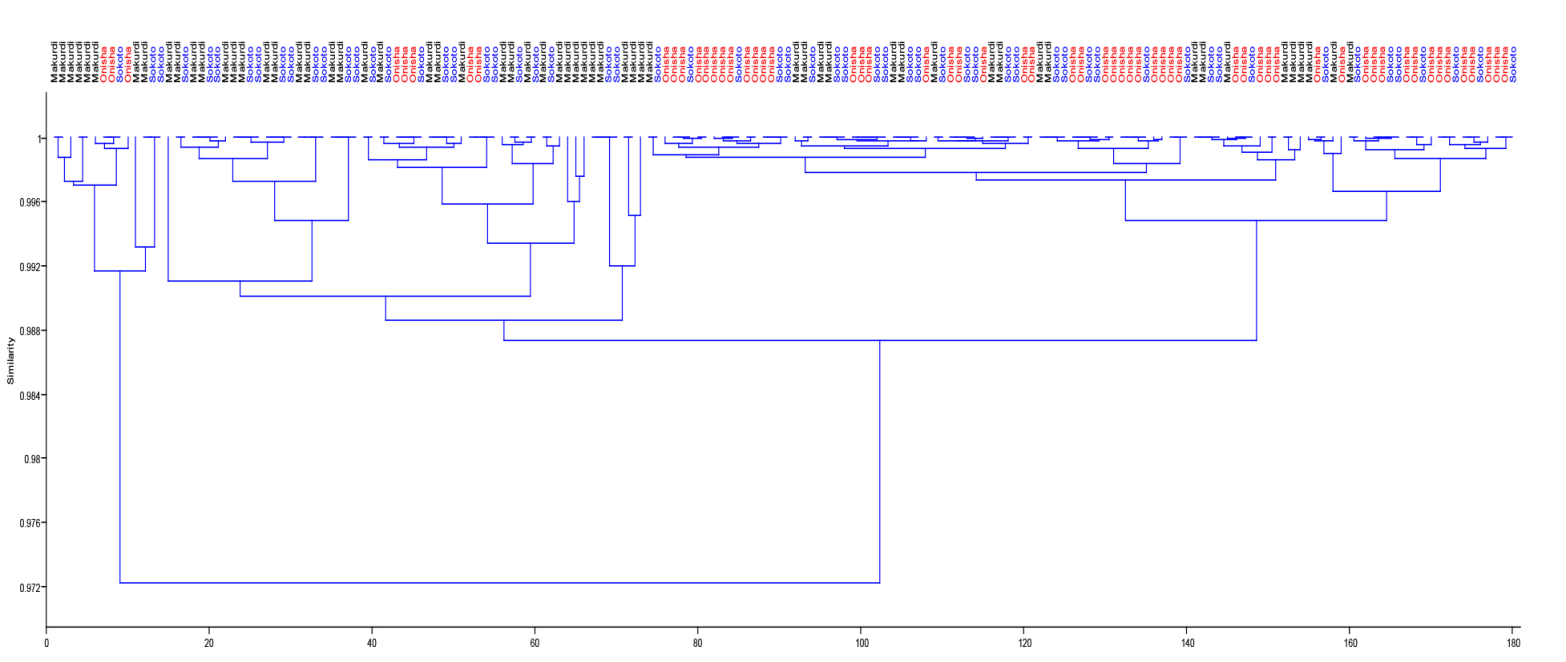
Dendrogram with complete linkage and euclidean distance for meristic count of *H. longifilis* from three geographical locations in Nigeria. The biplot shows individual fish scores. Black label = Makurdi; Red label = Onisha; Blue label = Sokoto.

The result of the univariate analysis of variance (ANOVA) of the meristic counts revealed significant differences in four of the five parameters (with the exception of PFRC). The Onitsha strain had significantly higher counts compared to the Sokoto population, while the least values were gotten from the Makurdi population (*P* < 0.05). The PCA for the transformed homologous meristic counts of the *H. longifilis* samples presented in Table 4 showed a cumulative variance of 76.22% for PC1 (55.51%) and PC2 (20.71%) as they had eigenvalue above unity (1). While the PC1 contained only positive coefficients, the PC2 had mixed positive and negative coefficients. The biplot shown in Fig. 4 further showed that the population could not be separated into unique multivariate spaces using the meristic counts, hence, influential variables would largely not be accurate. Also, the dendrogram of complete linkage and Euclidean distance shown in Fig. 5 showed multiple overlaps of the samples from the different ecoregions using the meristic counts.[Table t5-tlsr_35-1-161]

## DISCUSSION

The importance of obtaining detailed knowledge of the population structure of commercially exploited fish species cannot be overemphasised as it allows for the efficient management of the fisheries. It is the prerequisite for any genetic improvement programme if it is to be successful (Okomoda *et al*. 2022). Morphological characteristics such as morphometric parameters and meristic counts are commonly used to identify stocks of fish, populations, and species (Turan *et al*. 2004). It relies on the detection of subtle differences in shapes independent of size to discriminate populations within a given species. Generally, the variability observed or measured encompasses all aspects of the phenotypic variables (Tave 1993). Our study showed significant differences in 13 of the 39 morphometric parameters measured and in four of the five meristic counts (with the exception of PFRC). The study by Ajado and Edokpayi (2003) had earlier reported significant differences in the number of dorsal rays and the gill raker count in their study of two populations of the Clariid *Clarias gariepinus* population from Delta and Lagos. Similarly, Agbebi *et al*. (2009) reported significant differences in some parameters of *H. bidorsalis* with higher values linked to the population from Gboko compared to those from Onitsha and Jos. The differences in the reports of these studies could be linked to the difference in species, sizes and the number of samples used for the various studies.

Those intra-specific morphological differences noticeable in different population structures are usually not directly under the control of the genes but are subjected to environmental modification (Clay 1977). This is because fishes are the most susceptible vertebrate to environmentally induced morphological variations; hence, they demonstrate greater variance within and between populations than any other vertebrate (Solomon *et al*. 2015; Okomoda *et al*. 2018). Betiku (2006) suggested that the mechanism of action resulting from these includes quick adaptation and modification of physiological and behavioural states in response to environmental changes, therefore, modifying the fish’s morphology significantly. Hence, the non-discrimination of various populations in our current study into unique multivariate spaces (using the meristic and morphometric parameters) might be due to the low degree of environmental impact as well as the low level of adaptation of the population at the time of the study was conducted. This was demonstrated in our earlier study with different populations of wild and cultured *Anabas testudineus* in Malaysia (Okomoda *et al*. 2022). That previous study showed that the cultured population was unique and distinct, while an extensive overlap characterised the wild populations suggesting a similar origin of the stock. This, therefore, strengthens the narrative that the differences in the degree of environmental impact and level of adaptation of the population could have dictated the level of morphological plasticity observed in different fish species.

Due to the overlap of the different populations, this study could not suggest the most influential parameters for the discrimination of the fish stocks. This contrasts with the findings of Solomon *et al*. (2015) who suggested head length, BDA, and eye diameter as the most influential morphometric parameters for the discrimination of cultured and wild African catfish *C. gariepinus* in Nigeria. Haddon and Willis (1995) also stated that morphometrics measurements of the head and the body depth were the most important characters in the identification of the population of the Angler fish, *Lophius vorernus*, Pacific herring, *Clupea pallasi* and Orange roughy *Hoplostethus atlanticus*. Aside from the influence of the changes in environmental factors (Turan *et al*. 2005; Solomon *et al*. 2015); differences in geographical and ancestral origin (Hossain *et al*. 2010), phenotypic variation in natural stock sometimes reveals genetic adaptation to selection pressures (Solem *et al*. 2006). Therefore, the outcome of studies is most likely evidence of the possible combination of genetic and environmental factors as it influences the morphology of the fish (Olufeagba & Yisa 2003). Hence it is therefore important to initiate genetic studies of the fish from these three eco-regions to determine the levels of genetic variations despite the observable morphometric similarities.

## CONCLUSION

Although morphological characterisation has been reported in many previous studies to be useful in the discrimination of fish stock, the current study has demonstrated otherwise when considering three Nigerian populations (namely, Makurdi, Onisha, and Sokoto population). While the exact reason for this deviant observation may not be well understood, genetic studies are urgently needed to determine the level of variability among the population should selectively breeding of the fish for improvement be considered. Future studies can also consider the morphometric comparison of wild and cultured *H. longifilis* as many years of indiscriminate breeding could have led to unplanned backcrossing of hybrids with pure crosses.

## Figures and Tables

**Table 1:** Morphometric measurements of *H. longifilis* from three eco-regions in Nigeria. Numbers in each cell are means in centimeters (cm) ± standard error.

Variables	Makurdi	Onitsha	Sokoto	*P*-value
SL	40.44 ± 0.69	41.09 ± 0.70	40.70 ± 0.61	0.793
BD	5.24 ± 0.12	5.23 ± 0.11	5.15 ± 0.10	0.804
HL	11.32 ± 0.26	11.99 ± 0.22	11.85 ± 0.22	0.115
IOD	5.95 ± 0.14	5.89 ± 0.14	5.84 ± 0.12	0.831
ED	0.60 ± 0.02	0.59 ± 0.01	0.59 ± 0.01	0.688
PAD	22.50 ± 0.37	22.60 ± 0.33	22.32 ± 0.31	0.832
AFL	15.10 ± 0.30	15.12 ± 0.29	15.09 ± 0.26	0.996
AFH	2.10 ± 0.07	2.24 ± 0.06	2.16 ± 0.06	0.288
OFL	1.05 ± 0.03	1.01 ± 0.02	0.98 ± 0.02	0.113
OFW	0.74 ± 0.01	0.75 ± 0.02	0.74 ± 0.01	0.961
DBTOP	8.61 ± 0.12	8.61 ± 0.12	8.59 ± 0.13	0.992
PDL	14.51 ± 0.21	14.76 ± 0.21	14.61 ± 0.19	0.710
DFL	33.93 ± 0.99	34.85 ± 1.04	33.62 ± 0.95	0.670
DFH	3.28 ± 0.07	3.19 ± 0.07	3.26 ± 0.06	0.625
ADFTA	10.72 ± 0.24	11.02 ± 0.22	10.81 ± 0.21	0.647
PDFTA	10.09 ± 0.26	10.88 ± 0.29	10.34 ± 0.24	0.118
CFL	5.99 ± 0.12	5.93 ± 0.09	5.92 ± 0.11	0.875
CFW	5.01 ± 0.13	5.00 ± 0.09	4.99 ± 0.10	0.997
CPD	3.62 ± 0.09	3.61 ± 0.08	3.56 ± 0.08	0.875
CPL	0.77 ± 0.04^a^	0.60 ± 0.01^c^	0.68 ± 0.03^b^	0.001
PrPeL	8.99 ± 0.17^c^	9.86 ± 0.13^a^	9.49 ± 0.16^b^	0.001
PFW	2.83 ± 0.14^b^	3.56 ± 0.07^a^	3.09 ± 0.12^b^	0.001
PFDT	10.02 ± 0.17^b^	10.67 ± 0.12^a^	10.35 ± 0.16^ab^	0.011
PSL	4.08 ± 0.13^b^	4.63 ± 0.09^a^	4.37 ± 0.12^ab^	0.008
PFL	3.87 ± 0.10^b^	4.24 ± 0.07^a^	4.05 ± 0.09^ab^	0.027
PFW	2.21 ± 0.09	2.16 ± 0.06	2.15 ± 0.06	0.867
PrPL	19.35 ± 0.44	20.53 ± 0.21	20.22 ± 0.35	0.057
DFPF	3.77 ± 0.08^a^	3.42 ± 0.07^b^	3.53 ± 0.07^b^	0.005
LML	0.72 ± 0.03^a^	0.58 ± 0.01^b^	0.64 ± 0.02^b^	0.001
UML	1.96 ± 0.06	1.91 ± 0.03	1.97 ± 0.05	0.695
VL	3.91 ± 0.08	4.01 ± 0.04	3.92 ± 0.04	0.428
VW	4.92 ± 0.12^c^	5.66 ± 0.07^a^	5.37 ± 0.09^b^	0.128
VG	0.18 ± 0.005	0.17 ± 0.005	0.18 ± 0.005	0.980
SnL	3.55 ± 0.07^b^	3.79 ± 0.03^a^	3.71 ± 0.05^a^	0.007
PrML	0.51 ± 0.02^a^	0.39 ± 0.01^b^	0.43 ± 0.01^b^	0.001
PrMW	3.67 ± 0.13	3.50 ± 0.03	3.61 ± 0.08	0.420
MBL	14.17 ± 0.61^b^	17.25 ± 0.70^a^	16.26 ± 0.61^a^	0.003
NBL	7.56 ± 0.39^b^	9.49 ± 0.22^a^	9.07 ± 0.29^a^	0.001

*Notes:* Mean in the same row with different superscript differ significantly (*P* < 0.05). SL = Standard length, BD = Body depth, HL = Head Length, IOD = Inter-orbital distance, ED = Eye diameter, PAD = Pre anal distance, AFL = Anal fin length, AFH = Anal fin height, OFL = Occipital fontanelle length, OFW = Occipital fontanelle width, DBTOP = Distance between the occipital process and dorsal fin, PeFL = Pectoral fin length, DFL = Dorsal fin length, DFH = Dorsal fin height, ADFTA = Anterior dorsal fin to adipose fin, PDFTA = Posterior dorsal fin to adipose fin, CFL = Caudal fin length, CFW = Caudal fin width, CFD = Caudal peduncle depth, CFL = Caudal peduncle length, PrPeL = Prepectoral length, PeFW = Pectoral fin width, PFDT = Pelvic fin distance to anal fin, PSL = Pelvic spin length, PFL = Pelvic fin length, PFW = Pelvic fin width, PrPL = Prepelvic length, DFPF = Distances between dorsal fin end and adipose fin origin, LML = Lower mandibular length, UML = Upper mandibular length, VL = Vomerine length, VW = Vomerine width, VG = Vomerine gap, SnL = Snout length, PrML = Premaxillary length, PrMW = Premaxillary width, MBL = Maxillary barbell length, NBL = Nasal barbell length.

**Table 2:** Morphometric parameters of *H. longifilis* from three eco-regions in Nigeria expressed as percentages of standard length. Numbers in each cell are means in percentages (%) ± standard error.

Variables	Makurdi	Onisha	Sokoto	*P*-value
TL	115.50 ± 0.71	114.57 ± 0.57	114.67 ± 0.63	0.542
BD	12.97 ± 0.22	12.76 ± 0.21	12.68 ± 0.20	0.597
HL	28.01 ± 0.46	29.17 ± 0.21	29.16 ± 0.43	0.060
IOD	14.68 ± 0.16	14.34 ± 0.26	14.34 ± 0.19	0.406
ED	1.50 ± 0.04	1.46 ± 0.03	1.45 ± 0.03	0.503
PAD	55.79 ± 0.53	55.19 ± 0.49	55.09 ± 0.64	0.643
AFL	37.38 ± 0.40	36.86 ± 0.39	37.17 ± 0.48	0.712
AFH	5.19 ± 0.14	5.46 ± 0.11	5.30 ± 0.11	0.283
OFL	2.64 ± 0.09	2.49 ± 0.06	2.44 ± 0.06	0.113
OFW	1.87 ± 0.05	1.84 ± 0.05	1.85 ± 0.05	0.927
DBTOP	21.43 ± 0.25	21.08 ± 0.26	21.24 ± 0.31	0.685
PDL	36.08 ± 0.39	36.07 ± 0.36	36.15 ± 0.52	0.990
DFL	84.53 ± 2.52	85.41 ± 2.69	83.52 ± 2.63	0.879
DFH	8.12 ± 0.11	7.79 ± 0.13	8.06 ± 0.15	0.207
ADFTA	26.45 ± 0.31	26.79 ± 0.28	26.66 ± 0.45	0.813
PDFTA	24.88 ± 0.38	26.35 ± 0.42	25.42 ± 0.49	0.072
CFL	14.87 ± 0.23	14.49 ± 0.20	14.62 ± 0.24	0.514
CFW	12.46 ± 0.29	12.27 ± 0.26	12.37 ± 0.27	0.897
CPD	8.95 ± 0.15	8.79 ± 0.15	8.77 ± 0.16	0.676
CPL	1.91 ± 0.09^a^	1.37 ± 0.07^c^	1.63 ± 0.07^b^	0.001
PrPeL	22.29 ± 0.27^b^	24.32 ± 0.53^a^	23.61 ± 0.51^a^	0.008
PFW	6.94 ± 0.30^b^	8.76 ± 0.22^a^	7.69 ± 0.31^b^	0.001
PFDT	24.91 ± 0.33	26.32 ± 0.54	25.77 ± 0.53	0.126
PSL	10.05 ± 0.24^b^	11.40 ± 0.31^a^	10.84 ± 0.33^ab^	0.008
PFL	9.57 ± 0.19^b^	10.49 ± 0.28^a^	10.09 ± 0.28^ab^	0.050
PFW	5.45 ± 0.21	5.31 ± 0.15	5.37 ± 0.18	0.881
PrPL	47.87 ± 0.74	50.59 ± 0.99	50.41 ± 1.20	0.109
DFPF	9.34 ± 0.16^a^	8.45 ± 0.24^b^	8.78 ± 0.21^b^	0.010
LML	1.79 ± 0.09^a^	1.44 ± 0.06^b^	1.58 ± 0.05^b^	0.001
UML	4.84 ± 0.12	4.72 ± 0.11	4.91 ± 0.16	0.596
VL	9.75 ± 0.19	9.87 ± 0.18	9.75 ± 0.18	0.864
VW	12.16 ± 0.17^b^	13.89 ± 0.20^a^	13.35 ± 0.29^a^	0.001
VG	0.44 ± 0.02	0.43 ± 0.02	0.44 ± 0.01	0.850
SnL	8.82 ± 0.17^b^	9.35 ± 0.16^a^	9.24 ± 0.19^ab^	0.001
PrML	1.27 ± 0.06^a^	0.96 ± 0.03^b^	1.08 ± 0.04^b^	0.001
PrMW	9.08 ± 0.28	8.62 ± 0.14	8.99 ± 0.23	0.350
MBL	34.68 ± 1.18^b^	42.78 ± 1.97^a^	40.60 ± 1.70^a^	0.002
NBL	18.40 ± 0.74^b^	23.44 ± 0.71^a^	22.67 ± 0.86^a^	0.001

*Notes:* Mean in the same row with different superscript differ significantly (*P* < 0.05). TL = Total length, BD = Body depth, HL = Head length, IOD = Inter-orbital distance, ED = Eye diameter, PAD = Pre anal distance, AFL = Anal fin length, AFH = Anal fin height, OFL = Occipital fontanelle length, OFW = Occipital fontanelle width, DBTOP = Distance between the occipital process and dorsal fin, PDL = Predorsal length, DFL = Dorsal fin length, DFH = Dorsal fin height, ADFTA = Anterior dorsal fin to adipose fin, PDFTA = Posterior dorsal fin to adipose fin, CFH = Caudal fin height, CPD = Caudal peduncle depth, CFW = Caudal fin width, CPL = Caudal peduncle length, PrPEL = Prepectoral length, PeFW = Pectoral fin width, PFDT = Pelvic fin distance to anal fin, PSL = Pelvic spin length, PFL = Pelvic fin length, PFW = Pelvic fin width, PrPL = Prepelvic length, DFPF = Distances between dorsal fin end and adipose fin origin, LML = Lower mandibular length, UML = Upper mandibular length, VL = Vomerine length, VW = Vomerine width, VG = Vomerine gap, SnL = Snout length, PrML = Premaxillary length, PrMW = Premaxillary width, MBL = Maxillary barbell length, NBL = Nasal barbell length, ADFL = Adipose fin length, HW = Head width, PeFL = Pectoral fin length, PPcL = Prepectoral length, PFH = Pelvic fin height, AFRN = Anal fin ray number, PvFAF = Pelvic fin to anal fin, CFRN = Caudal fin ray number, PmxL = Premaxillary length, DFRN = Dorsal fin ray number, CFL = Caudal fin length.

**Table 3:** Meristic counts of the different sexes of *H. longifilis* from three eco-regions in Nigeria. Numbers in each cell are means in percentages (%) ± standard error.

Variables	Makurdi	Onisha	Sokoto	*P*-value
AFRC	47.50 ± 0.57^c^	52.20 ± 0.34^a^	49.68 ± 0.57^b^	0.001
CFRC	19.08 ± 0.21^b^	20.53 ± 0.17^a^	19.60 ± 0.22^b^	0.001
DFRC	36.55 ± 0.67^c^	41.37 ± 0.48^a^	38.47 ± 0.67^b^	0.023
PFRC	5.92 ± 0.12	5.95 ± 0.07	5.83 ± 0.10	0.706
PFRC	7.55 ± 0.29^c^	10.63 ± 0.14^a^	8.62 ± 0.34^b^	0.001

*Note*: Mean in the same row with different superscript differ significantly (*P* < 0.05). AFRC = Anal fin ray count, CFRC = Caudal fin ray count, DFRC = Dorsal fin ray count, PFRC = Pelvic fin ray count, PeFRC = Pectoral fin ray count.

**Table 4:** Principal component analysis of transformed morphometric measurements of *H. longifilis* from three eco-regions in Nigeria (*n* = 162). Values in the body of the table are component loading.

Variable	PC1	PC2	PC3
TL	−0.15	0.21	−0.14
BD	−0.15	0.25	−0.04
HL	−0.09	0.25	0.16
IOD	−0.06	0.01	0.07
ED	−0.15	0.14	−0.07
PAD	−0.20	0.27	−0.13
AFL	−0.16	0.35	−0.06
AFH	−0.06	−0.04	0.23
OFL	−0.11	−0.08	−0.16
OFW	−0.16	−0.10	−0.13
DBTOP	−0.12	−0.09	−0.07
PDL	−0.21	0.23	−0.09
DFL	−0.14	0.14	−0.08
DFH	−0.11	0.04	0.00
ADFTA	−0.12	0.39	0.01
PDFTA	−0.07	0.37	0.10
CFL	−0.15	−0.01	0.03
CFW	−0.14	−0.03	−0.05
CPD	−0.09	−0.02	0.04
CPL	−0.04	−0.08	−0.34
PrPeL	−0.23	−0.15	0.13
PFW	−0.17	−0.15	0.16
PFDT	−0.25	−0.12	0.07
PSL	−0.19	−0.13	0.15
PFL	−0.22	−0.21	0.14
PFW	−0.16	−0.03	0.02
PrPL	−0.25	−0.08	0.05
DFPF	−0.16	−0.15	−0.16
LML	−0.08	−0.12	−0.40
UML	−0.22	−0.09	−0.11
VL	−0.22	−0.09	−0.08
VW	−0.22	−0.01	0.22
VG	−0.05	−0.16	−0.09
SnL	−0.23	−0.05	0.15
PrML	−0.06	−0.05	−0.45
PrMW	−0.17	−0.04	−0.24
MBL	−0.20	−0.09	0.17
NBL	−0.20	−0.03	0.14
Eigenvalue	10.83	3.58	3.16
% of variance	28.49	9.43	8.33
Cumulative % variance	28.49	37.92	46.25

*Notes:* TL = Total length, BD = Body depth, HL = Head length, IOD = Inter-orbital distance, ED = Eye diameter, PAD = Pre anal distance, AFL = Anal fin length, AFH = Anal fin height, OFL = Occipital fontanelle length, OFW = Occipital fontanelle width, DBTOP = Distance between the occipital process and dorsal fin, PDL = Predorsal length, DFL = Dorsal fin length, DFH = Dorsal fin height, ADFTA = Anterior dorsal fin to adipose fin, PDFTA = Posterior dorsal fin to adipose fin, CFH = Caudal fin height, CPD = Caudal peduncle depth, CFW = Caudal fin width, CPL = Caudal peduncle length, PrPEL = Prepectoral length, PeFW = Pectoral fin width, PFDT = Pelvic fin distance to anal fin, PSL = Pelvic spin length, PFL = Pelvic fin length, PFW = Pelvic fin width, PrPL = Prepelvic length, DFPF = Distances between dorsal fin end and adipose fin origin, LML = Lower mandibular length, UML = Upper mandibular length, VL = Vomerine length, VW = Vomerine width, VG = Vomerine gap, SnL = Snout length, PrML = Premaxillary length, PrMW = Premaxillary width, MBL = Maxillary barbell length, NBL = Nasal barbell length, ADFL = Adipose fin length, HW = Head width, PeFL = Pectoral fin length, PPcL = Prepectoral length, PFH = Pelvic fin height, AFRN = Anal fin ray number, PvFAF = Pelvic fin to anal fin, CFRN = Caudal fin ray number, PmxL = Premaxillary length, DFRN = Dorsal fin ray number, CFL = Caudal fin length.

**Table 5 t5-tlsr_35-1-161:** Principal component analysis of meristic counts of *H. longifilis* from three eco-regions in Nigeria (*n* = 162). Values in the body of the table are component loading.

Variable	PC1	PC2
AFRC	0.53	−0.07
CFRC	0.46	−0.12
DFRC	0.49	0.19
PFRC	0.08	−0.96
PeFRC	0.51	0.16
Eigenvalue	2.78	1.04
% of variance	55.51	20.71
Cumulative % variance	55.51	76.22

*Notes:* AFRC = Anal fin ray count, CFRC = Caudal fin ray count, DFRC = Dorsal fin ray count, PFRC = Pelvic fin ray count, PeFRC = Pectoral fin ray count.
